# No such thing as a perfect hammer: comparing different objective function specifications for optimal control

**DOI:** 10.1007/s10100-016-0446-7

**Published:** 2016-06-22

**Authors:** D. Blueschke, I. Savin

**Affiliations:** 1 Alpen-Adria-Universität Klagenfurt, Klagenfurt, Austria; 2 Chair for Economic Policy, Karlsruhe Institute of Technology, Karlsruhe, Germany; 3 Bureau d’Economie Théorique et Appliquée, UMR 7522, CNRS, Université de Strasbourg, Strasbourg, France; 4 Faculty of Economics and Business Administration, Friedrich Schiller University of Jena, Jena, Germany; 5 Chair for Econometrics and Statistics, Graduate School of Economics and Management, Ural Federal University, Yekaterinburg, Russian Federation

**Keywords:** Differential evolution, Nonlinear optimization, Optimal control, Least median of squares, C54, C61, E27, E61, E63

## Abstract

Linear-quadratic (LQ) optimization is a fairly standard technique in the optimal control framework. LQ is very well researched, and there are many extensions for more sophisticated scenarios like nonlinear models. Conventionally, the quadratic objective function is taken as a prerequisite for calculating derivative-based solutions of optimal control problems. However, it is not clear whether this framework is as universal as it is considered to be. In particular, we address the question whether the objective function specification and the corresponding penalties applied are well suited in case of a large exogenous shock an economy can experience because of, e.g., the European debt crisis. While one can still efficiently minimize quadratic deviations around policy targets, the economy itself has to go through a period of turbulence with economic indicators, such as unemployment, inflation or public debt, changing considerably over time. We test four alternative designs of the objective function: a least median of squares based approach, absolute deviations, cubic and quartic objective functions. The analysis is performed based on a small-scale model of the Austrian economy and illustrates a certain trade-off between quickly finding an optimal solution using the LQ technique (reaching defined policy targets) and accounting for alternative objectives, such as limiting volatility in economic performance. As an implication, we argue in favor of the considerably more flexible optimization technique based on heuristic methods (such as Differential Evolution), which allows one to minimize various loss function specifications, but also takes additional constraints into account.

## Introduction

Today, several countries in the European Union face difficulties in mitigating their public budget deficit and debt issues, which were triggered by the last economic crisis. In 2010, for example, the first bail-out program for Greece (of 110 Billion Euro) was approved by the Troika of the International Monetary Fund, the European Central Bank and the European Commission. In 2013, a 47.5 % haircut for deposits above 100 Thousand Euro was applied to several Cypriot banks.

For the Austrian economy (and other countries of the Euro zone) such an event has a one-time (nonrecurring) negative impact on the budget balance. The question for the local government is how to react to such a budget balance shock. The optimal control framework is a well-known tool to address such a fiscal policy question (see, e.g., Feichtinger and Hartl 1986; Neck et al. 2008; Neck 2009). A ‘traditional’ way to consider optimal control problems is the linear quadratic (LQ) optimization technique. This technique is mainly based on works by Pontryagin et al. (1962) and Bellman (1957). There are several more sophisticated numerical algorithms, which are also based on the LQ optimization framework, and allow us to consider nonlinear problems as well, e.g., the OPTCON algorithm developed by Blueschke-Nikolaeva et al. (2012). However, a characteristic property of the LQ framework is its sensitivity to outliers. The objective (or loss, as it will be referred henceforth) function is formulated in a quadratic way. A squared outlier influences the objective function considerably, forcing an active use of control variables, which might be undesirable in certain situations. For example, Fatas and Mihov (2003) show that an aggressive use of fiscal policy induces significant macroeconomic instability. Thus, in case of a large exogenous shock, a policy maker faces an additional task of mitigating the effects of this shock without putting the stability of the whole system at risk.

In a recent study by Blueschke et al. (2013), a new way of handling optimal control problems is proposed. The authors test an evolutionary approach for this purpose, namely Differential Evolution (DE, Storn and Price 1997), which does not rely on the LQ framework. Blueschke et al. (2013) apply DE to optimal control problems in nonlinear dynamic economic systems with an asymmetric objective function where the ‘traditional’ OPTCON algorithm does not work. The application of the DE method increases computation time substantially but gives much more flexibility in designing the objective function and different system constraints.^1^ In the present paper we aim to use this flexibility of the DE method by introducing and solving an optimal control problem with different specifications of the objective function. In particular, we test four alternative designs of the loss function: a least median of squares based approach (LMS), absolute deviations, cubic and quartic objective functions.

The least median of squares (LMS) estimator (Rousseeuw 1984) is among the best known robust estimators for linear problems. It has been widely used in numerous applications of finance and technology (see, for example, Winker et al. 2011), and is regarded as a standard technique for the robust data analysis. It was demonstrated, however, that this function exhibits multiple equilibria search space, which is usually very hard to handle in ‘traditional’ optimal control problems (see, e.g., Feichtinger 2004), but can be efficiently solved using the DE approach. The main advantage in minimizing the median of squares instead of the mean is the robustness, or rather non-sensitivity, of the LMS framework to unique large outliers. We apply this framework to design the objective function in an optimal control problem and check how LMS behaves in this case. The question that we address here is whether LMS-style-shaped objective function serves the goal of mitigating instability due to a one-time shock (which can be interpreted as an outlier) or this approach may even increase the volatility of the resulting states and controls obtained by the optimal control exercise.

An alternative objective function specification may be to apply deviations in states and controls to a power different from two (power two corresponds to the LQ framework). Simple intuition suggests that a power above two would penalize any deviation more, limiting volatility in states and controls generated by the exogenous shock. In particular, we consider the cubic and quartic penalties, which have not yet been addressed well in the literature.^2^ In addition, to get a more comprehensive understanding of how different penalties’ exponents drive the resulting optimal paths, absolute deviations (i.e. power one) are also considered.^3^

The rest of the paper is structured as follows. In Sect. 2 we describe a model of the Austrian economy experiencing an exogenous shock and solve it using the well known LQ framework. Section 3 contains a detailed description of alternative objective function specifications. Section 4 briefly reviews OPTCON and DE as different strategies for solving optimal control problems. Section 5 presents the results of the comparative study. Section 6 discusses the trade-off between alternative economic policy objectives and the corresponding loss function specifications, and concludes.

## The ATOPT model

In our study we consider a small nonlinear macroeconometric model of the Austrian economy (ATOPT). The ATOPT model can be seen as an extended Phillips curve connected with a simple model of the public finance sector. The model includes four state variables (the inflation, the unemployment rate, the budget balance and the public debt), one exogenous non-controlled variable and eight unknown (estimated) parameters. It includes one fiscal policy instrument, the primary balance, which allows a policy maker to control the whole system. Furthermore, it includes a channel for an external shock acting on the budget balance. The annual data for the time periods 1987–2013 yield 36 observations.^4^ The start period for the optimization is 2014 and the end period is 2023 (10 years).

*Model equations*:

(Standard deviations are given in parentheses)

1 $$\begin{aligned} pi_t= & {} \underset{(0.27)}{-0.14} + \underset{(0.10)}{0.60}*pi_{t-1} + \underset{(1.67)}{5.48}*\dfrac{1}{ur_t}, \end{aligned}$$

2 $$\begin{aligned} ur_t= & {} \underset{(0.34)}{6.58} \underset{(0.04)}{-0.11}*gr\_exr_t + \underset{(0.16)}{0.72}*prim\_balance_t, \end{aligned}$$

3 $$\begin{aligned} budget\_balance_t= & {} \underset{(0.15)}{-2.65} + \underset{(0.11)}{0.69}*prim\_balance_t + bb\_shock_t, \end{aligned}$$

4 $$\begin{aligned} debt_t= & {} debt_{t-1} + budget\_balance_t. \end{aligned}$$

Equations (1) and (2) can be regarded as an extended Phillips curve including a non-linear influence of the unemployment rate (denoted by ‘*ur*’) on the inflation rate (‘*pi*’). The unemployment rate^5^ is mainly driven by exogenous indicators: The growth rate of exports (‘$gr\_exr$’)^6^ and the fiscal policy of the national government. For the latter, the estimated model suggests an expansionary effect of the fiscal policy. The primary balance is the fiscal policy instrument, which is under direct control of the Austrian government. In contrast, the budget deficit (or surplus if positive) (denoted by ‘$budget\_balance$’) is estimated on the basis of the primary balance as stated in Eq. (3). Furthermore, the budget balance can be influenced directly by an exogenous shock (‘$bb\_shock_t$’). In our study, we apply a negative shock, which increases the budget deficit. Finally, the changes in the public debt level (‘*debt*’) are driven by the budget deficits (or surpluses if positive), as given in Eq. (4).

In the present study, the exogenous shock is modeled *ex ante* as if the government knew what a budget balance shock it would face in a few years time due to the European debt crisis. This is done mainly for simplicity to speed up the calculation and in order not to concentrate on the explanatory part of the model, given its limitations.^7^

The ATOPT model, as stated in Eqs. (1)–(4), captures a highly aggregated dynamics of the Austrian economy. We are aware that this is not sufficient to get accurate insights into the economic and/or fiscal situation in Austria. Instead, we use this model to test the performance of proposed approaches in case of a one-time shock which increases the budget deficit. The initial values, the target values and the weights of the variables considered in the objective function are reported in Table 1.

**Table 1:** Objective variables in the ATOPT model

Variable	Initial value	Target value	Weight
*pi*	1.6	2	1
*ur*	7.6	6	1
$budget\_balance$	$-$1.5	0	1
*debt*	74.5	74.5$\searrow$60	0.2
$prim\_balance$	0.7	0	1

The symbol $\searrow$ indicates that the target values for the objective variable *debt* are calculated in a linear decreasing way starting at initial value 74.5 and reaching the value 60 at the end of the planning horizon

### Nonlinear quadratic optimal control

In the first step we consider a standard optimum control problem with a quadratic objective function (a loss function to be minimized) and a nonlinear multivariate discrete-time dynamic system. The inter-temporal objective function is formulated in quadratic tracking form, which is often used in applications of optimal control theory to econometric models. ‘Traditionally’ it can be written as

5 $$\begin{aligned} J= \sum ^T_{t=1}L_t(x_t,u_t) \end{aligned}$$

with

6 $$\begin{aligned} L_t(x_t,u_t)=\frac{1}{2}\left( \begin{array}{c} x_t-\tilde{x}_t\\ u_t-\tilde{u}_t\\ \end{array}\right) 'W_t\left( \begin{array}{c} x_t-\tilde{x}_t\\ u_t-\tilde{u}_t\\ \end{array}\right) , \end{aligned}$$

where $x_t$ is an *n*-dimensional vector of state variables that describes the state of the economic system at any point in time *t*, $u_t$ is an *m*-dimensional vector of control variables, $\tilde{x}_t\in R^n$ and $\tilde{u}_t\in R^m$ are given ‘ideal’ (target) levels of the state and control variables respectively. *T* denotes the terminal time period of the finite planning horizon. $W_t$ is an $((n+m)\times (n+m))$ matrix specifying the relative weights of the state and control variables in the objective function. The $W_t$ matrix may also include a discount factor $\alpha$, $W_t=\alpha ^{t-1}W$. $W_t$ (or *W*) is symmetric. The dynamics of the system is given by ATOPT model as stated in Eqs. (1)–(4).

**Table 2:** Results for the ATOPT model with different settings

	OPTCON2		Differential evolution				
	Uncontrolled	Optimal	LMS	ABS	LQ	CUB	QUART
**No shock**							
J	693.20	188.91	126.36	73.75	188.91	2021.34	4437.39
std	n/a	n/a	(0.0000)	(0.0000)	(0.0003)	(0.0006)	(0.0009)
cpu	.001s	.5s	487s	472s	99s	310s	284s
LQJ	n/a	188.91	240.39	547.45	188.91	212.61	208.92
WVar	n/a	22.71	138.62	178.55	22.71	18.37	11.54
**With shock**							
J	1076.56	291.43	207.25	91.95	291.43	3760.97	9776.78
std	n/a	n/a	(0.0000)	(0.0000)	(0.0007)	(0.0010)	(0.0022)
cpu	.001s	.5s	665s	521s	116s	316s	315s
LQJ	n/a	291.43	334.71	884.78	291.43	321.96	320.05
WVar	n/a	72.50	198.52	304.38	72.50	62.90	56.73

Results for *LQJ* (linear-quadratic *J*) for alternative objective function specifications are obtained by re-evaluating the identified optimal sets of states and controls with the standard LQ function. Results on *WVar* (weighted variance) are calculated by estimating variance in the distribution of the states and controls obtained and accounting for the weights of the variables in the optimal control exercise as given in Table 1

In order to solve the stated nonlinear optimal control problem, the OPTCON algorithm (Blueschke-Nikolaeva et al. 2012) is used. This algorithm allows for a numerical approximation of a nonlinear solution based on the standard techniques of linear quadratic optimization (LQ).

### Optimal quadratic control in case of a one-time shock

We apply the nonlinear quadratic framework as stated in the previous section to calculate the optimal fiscal policy for the Austrian economy in presence of an external one-time shock on the budget balance. The start period for the optimization is 2014 and the end period is 2023. We assume the shock to occur in 2016. As we are modelling a negative shock, we set the variable $bb\_shock$ [see Eq. (3)] defining the government budget balance) to be −7 in period 3, i.e. $bb\_shock_3=-7$. Thus, it is assumed that due to the exogenous shock the budget balance of the Austrian economy worsens by 7 % points in 2016.

**Fig. 1:**
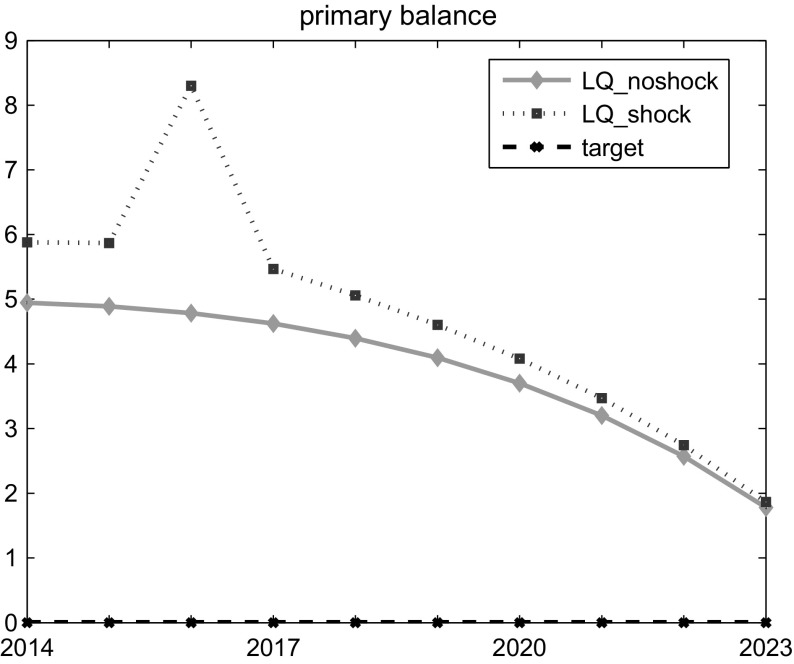
Control variable (LQ solution)

**Fig. 2:**
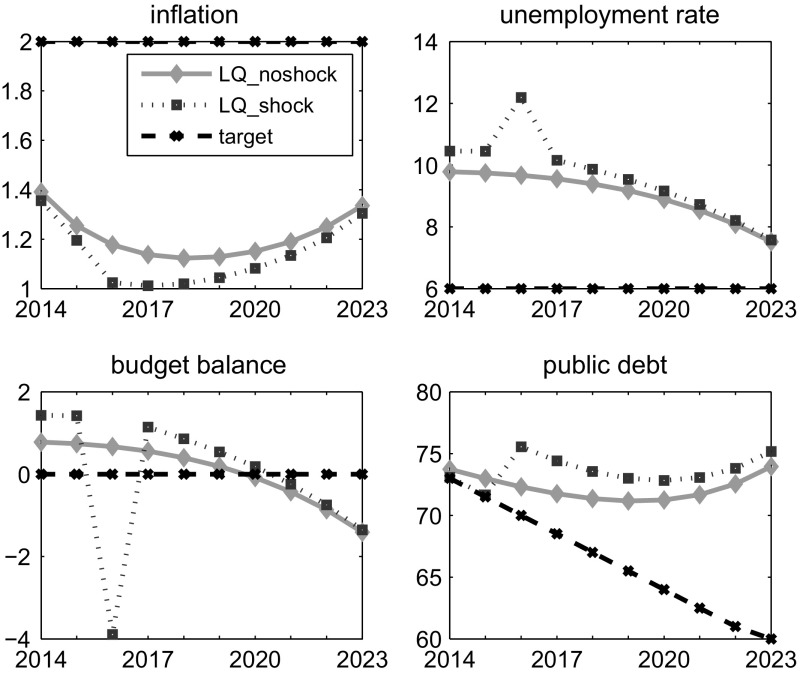
State variables for ATOPT (LQ solution)

We present here the optimal solution in situations with and without external shock, in order to show the effects of such a negative event to the Austrian economy. The results are obtained by the OPTCON algorithm but are identical for the Differential Evolution approach (see Sect. 4 for the description of the methods and Table 2 for the summary of the results). Figure 1 illustrates the optimal path for the control variable (primary balance), while Fig. 2 presents the optimal paths for the state variables (the rate of inflation, the unemployment rate, the government budget balance and the public debt).

Graphical results show a strong trade-off between fiscal stability and output oriented policy, which goes in line with the ‘philosophy’ of the ATOPT model. In the experiment without shock, the LQ approach requires a restrictive fiscal policy to be run in order to stabilize the financial situation. The policy is required to be more active at the beginning of the planning horizon ($prim\_balance_1=4.9$), continuously decreasing until the end ($prim\_balance_{10}=1.8$).

In the scenario with the shock, the LQ approach requires an even more restrictive fiscal policy to be run in the ‘shocked’ period, as an attempt to compensate for the negative impact of the shock ($prim\_balance_3=8.3$ and $budget\_balance_3 = {-}3.9$ vs. $bb\_shock_3= {-}7$). In order to measure the volatility of such a discretionary policy, we calculate a weighted variance (*WVar*) of the time series involved. In particular, after calculating the variances of all the specific variables (both the control and the states), we aggregate them into one indicator, using the weights applied in the earlier stage of optimization. This has the advantage of accounting for each variable differently (given that they have different units of measurement and, as a result, different orders of variance are involved), but at the same time not using any other arbitrary weights, which would increase the number of parameters affecting the results.^8^

*WVar* in the non-shocked scenario for the LQ framework is 22.71 and rises to 72.50 in the shocked scenario. The LQ framework is more or less forced to require a very active fiscal policy, due to quadratic costs of the outlier event. Such an intensive and restrictive fiscal policy has a relatively strong negative impact on the economic situation, with the unemployment rate rising by more than 2 % points in one period.

In the next section we test alternative forms of objective function specification. Special attention should be paid to the excess volatility of fiscal policy.

## Different objective function specifications

In this section, we describe four alternative objective function specifications for an optimal control framework. The proposed alternatives include an experiment using the idea of least median of squares (LMS), absolute deviations (ABS), a cubic objective function (CUB) and a quartic objective function (QUART).

### Least median of squares

We reformulate the objective function ‘J’ (Eqs. 5, 6) using the median of squares instead of the sum of squares on the corresponding states but not the control(s).^9^ The intuition behind the usage of a method like LMS is that, being more robust, it will devote little attention to the external shock, thus making the framework more stable to external effects. In particular, one can expect the volatility of the optimal paths of the states and controls to be lower using LMS than without it. As a result we get the following objective function:

7 $$\begin{aligned}&\displaystyle J=\sum ^N_{i=1}median(L^{x_i}_1,L^{x_i}_2,\ldots , L^{x_i}_T)\cdot T + \sum ^T_{t=1}L^u_t, \end{aligned}$$

8 $$\begin{aligned}&\displaystyle L^x_t(x_t)=\frac{1}{2}\left( x_t-\tilde{x}_t\right) 'W^x_t\left( x_t-\tilde{x}_t\right) , \end{aligned}$$

9 $$\begin{aligned}&\displaystyle L^u_t(u_t)=\frac{1}{2}\left( u_t-\tilde{u}_t\right) 'W^u_t\left( u_t-\tilde{u}_t\right) . \end{aligned}$$

$L^x_t$ represents the squared deviations between the state variables and their target values. $L^u_t$ represents the squared deviations between the control variables and their target values. As stated above and given in Eq. (7), the objective function for states is calculated as a median (over time) of squares (corresponds to the LMS approach). The control variables are handled in a traditional way in the objective function by summing the squares (corresponds to LQ framework). The difference in levels between $L^x$ and $L^u$ needs to be adjusted by factor *T* as given in Eq. (7).

### Absolute values

We calculate the objective function based on normal (non-quadratic or rather power equal to one) deviations. In order to prevent the problem of offsetting positive and negative numbers, the absolute values of the calculated deviations are used.

10 $$\begin{aligned}&\displaystyle J=\sum ^T_{t=1}L^x_t + \sum ^T_{t=1}L^u_t, \end{aligned}$$

11 $$\begin{aligned}&\displaystyle L^x_t(x_t)=\frac{1}{2}\left| x_t-\tilde{x}_t\right| 'W^x_t, \end{aligned}$$

12 $$\begin{aligned}&\displaystyle L^u_t(u_t)=\frac{1}{2}\left| u_t-\tilde{u}_t\right| 'W^u_t. \end{aligned}$$

### Cubic objective function

In this scenario the deviations from the targets are penalized by factor three. Similar to the previous scenario, we use the absolute values of deviations before calculating the exponent in order to prevent the problem of offsetting positive and negative numbers.

13 $$\begin{aligned} J= \sum ^T_{t=1}L_t(x_t,u_t), \end{aligned}$$

with

14 $$\begin{aligned} L_t(x_t,u_t)=\frac{1}{2}\left( \left( \begin{array}{c} |x_t-\tilde{x}_t|\\ |u_t-\tilde{u}_t|\\ \end{array} \right) '\right) ^{1.5}W_t\left( \left( \begin{array}{c} |x_t-\tilde{x}_t|\\ |u_t-\tilde{u}_t|\\ \end{array} \right) \right) ^{1.5}. \end{aligned}$$

### Quartic objective function

Finally, we calculate the deviations between state variables and the corresponding target paths to the power four.

15 $$\begin{aligned} J= \sum ^T_{t=1}L_t(x_t,u_t), \end{aligned}$$

where

16 $$\begin{aligned} L_t(x_t,u_t)=\frac{1}{2}\left( \left( \begin{array}{c} x_t-\tilde{x}_t\\ u_t-\tilde{u}_t\\ \end{array} \right) '\right) ^2W_t\left( \left( \begin{array}{c} x_t-\tilde{x}_t\\ u_t-\tilde{u}_t\\ \end{array} \right) \right) ^2. \end{aligned}$$

## Optimization algorithms

### OPTCON

The OPTCON algorithm determines approximate solutions to optimal control problems with a quadratic objective function and a nonlinear multivariate dynamic system. It relies on the standard techniques of the LQG framework^10^ and combines elements of previous algorithms developed by Chow (1975, (1981) and Kendrick (1981). In our experiments we use the latest version of the OPTCON algorithm, which is called OPTCON2. We skip the presentation of the OPTCON algorithm, which can be found in more detail in Blueschke-Nikolaeva et al. (2012) and Savin and Blueschke (2015).

### Differential evolution

Proposed by Storn and Price (1997), Differential Evolution (DE) is a population based optimization technique for continuous objective functions. DE belongs to the so-called heuristic optimization approaches [for an overview of these techniques see Gilli and Winker (2009)] and is well acknowledged to be efficient at exploring complex search spaces with multiple local minima, but also for being relatively easy to apply, as it needs little parameter tuning. For applications of DE in finance, risk management and innovation management see Lyra et al. (2010), Winker et al. (2011) and Egbetokun and Savin (2014) respectively. Applications of DE, but also other heuristics (such as particle swarm optimization), can be also found in the area of optimal control, but mainly in the field of engineering (Cruz et al. 2003; Modares and Sistani 2011).

A detailed description on how DE deals with an optimal control problem for a deterministic scenario (single parameter set) can be found in Blueschke et al. (2013, pp. 824–825), while Savin and Blueschke (2015, pp. 7–10) extend the exercise for the stochastic scenario. For the sake of this study it suffices to say that starting with an initial population of random solutions (line 2 in Algorithm 1), DE updates this population by linear combination (line 7: with the scale factor *F* determining the shrinkage rate in exploring the search space) and crossover (line 9: with *CR* standing for the crossover rate) of four different solution vectors into one, and selects the fittest solutions among the original and the updated population. This continues until some stopping criterion is met. Each member of the population (each candidate solution) contains all control variables for all time periods. Thus, each candidate $i=1,\ldots ,p$ represents an alternative complete solution path for the whole optimum control problem, and is given as an ($m\times T$)-matrix $P_{.,.,i}^{(1)}=(P_{j,t,i}^{(1)})_{\mathop {{j=1, \ldots , m}_{t=1,\ldots ,T}}} = (u^{(1),i}_1, u^{(1),i}_2, \ldots , u^{(1),i}_T)$, where $u^{(1),i}_t$ is an *m*-dimensional vector of controls and *T* is the size of the planning horizon.

It is important to mention that each candidate solution is also described by the time paths of corresponding state variables, which results from the dynamic system *f*, parameter set $\theta$ and the selected controls, i.e. ($x^{(1),i}_{t=1,\ldots ,T}=f(\ldots ,u^{(1),i}_{t=1,\ldots ,T},\theta ,\ldots )$). For each candidate solution (for each set of control variables) and for each parameter set $\theta$, there is a unique set of state variables. These state variables are not directly included in a candidate solution but they contribute to the objective function to be minimized.
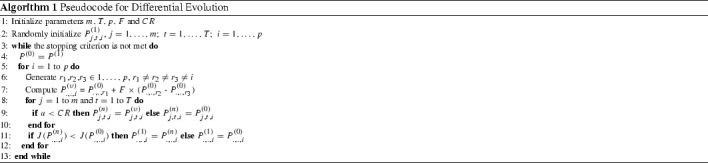


Tuning the DE parameters is a problem specific exercise. For this reason we conducted a series of simulation experiments, as described in Blueschke et al. (2013, pp. 825–826), to calibrate the DE parameters. In brief, we first fix *F* and *CR* both to be equal to 0.55 (average value), and test different population sizes (between 5*d* and 30*d*), increasing the number of generations *g* until DE results in the same outcome for several replications. Having defined the population size *p*, we then run DE for different *CR* and *F* ranging between 0.1 and 1. We identify combinations of parameter values with the lowest average number of generations required to achieve the value-to-reach (*VTR*), which is set to 101 % of the best objective value achieved by DE over the one hundred parameter combinations considered. It turns out that for the LMS objective function the optimal combination of parameters is $F=0.5$ and $CR=0.8$ with the population size $p=50\times m\times T$ (where *m* is the number of controls and *T* is the number of time periods involved), while the number of DE generations $g^{max} =2500$. These parameters are taken sufficiently large to ensure convergence. For the remaining objective function specifications, which are apparently simpler to solve, we set the following parameters: $F=0.4$, $CR=0.1$, $p=10\times m\times T$, while $g^{max} =750$. Additionally, we check the convergence within the population by looking at the candidates’ objective values. Working on a continuous optimization problem, it is unlikely that two candidates reach exactly the same value, but a difference of 0.0001 % between the fittest solution and a few closest followers is realistic and is therefore applied in our study. Thus, the DE algorithm stops if 30 % of the solutions in the population reach this deviation from the best solution available. In addition, if for 100 generations more than 50 % of the solutions in the population do not improve, the algorithm also stops. For each objective function DE is restarted ten times.

Both methods, OPTCON and DE, are implemented in Matlab to simplify their comparison. The corresponding computational time for the different objective functions tested in this study necessarily varies depending on the complexity of a particular problem (largest for LMS and absolute deviations), but never exceeds 12 min using Matlab 8.5.0 and a Pentium IV 2.7 GHz.

## Optimal control results

The four presented objective function alternatives and the nonlinear dynamic system given by the ATOPT model constitute four different optimal control problems solved using the OPTCON and the Differential Evolution methods as described above.

**Fig. 3:**
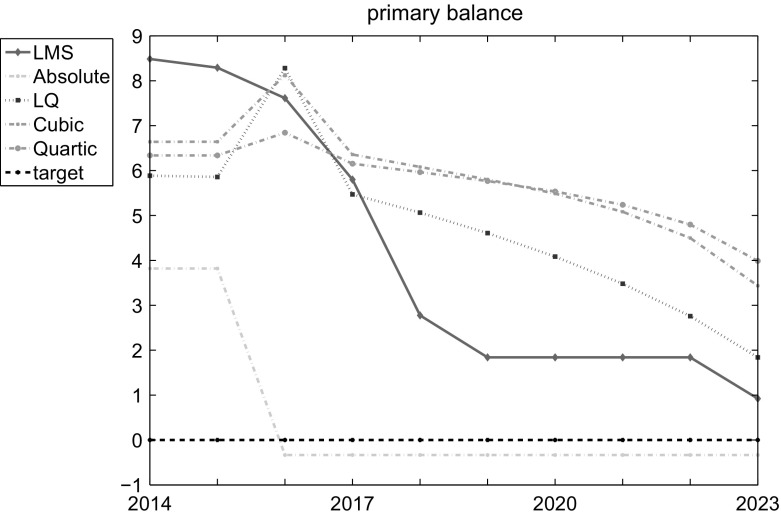
Optimal paths of control variable

**Fig. 4:**
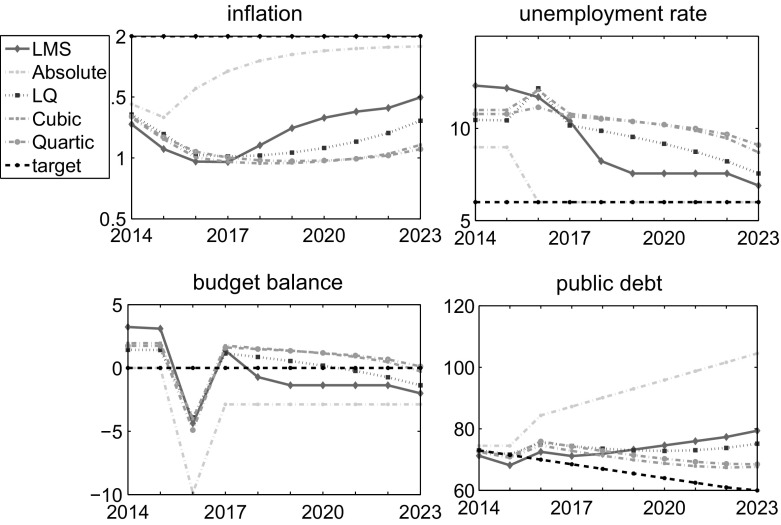
Optimal paths of state variables

Figures 3 and 4 show the optimal results for the four proposed objective function specifications in the presence of a one-time shock. The LQ approach requires an extremely restrictive fiscal policy to be run in the third period (2016) to try to compensate the negative impact of the shock. The LMS approach allows smoothing of the effects of the one-time shock but increases the overall volatility and significantly differs from the optimal path both in control and state variables. Thus, the robust characteristics of the original LMS approach do not hold for optimal control problems.

The ABS scenario produces results, which deviate dramatically from other alternatives. Considering the absolute deviations in the objective functions makes it possible to give much less importance to the one-time shock but it also ignores larger deviations in states. In such a case, the government is required to run an extremely expansionary fiscal policy with strong positive effects on unemployment but drastic consequences for public finance, with the public debt exceeding 100 % of GDP at the end of the planning horizon.

The cubic and quartic objective functions, in contrast, restrict the volatility of the optimal paths of the corresponding states and controls, thus constituting a more robust way a policy maker can respond to an exogenous shock. The reason is that the penalty rises exponentially for any deviation from the targets stated, so that the effects of the shock are absorbed by a larger number of periods. Clearly, the larger the exponent of the penalty, the smoother the paths of the corresponding variables.

Table 2 summarizes the results for the LQ framework and its four alternatives in terms of:

- Minimal objective function values *J* achieved. These values are estimated by applying different function specifications and respective penalties, making them incomparable with each other;
- Standard deviation in DE results over restarts and cpu time required to obtain the results (per restart);
- Minimal objective function values estimated with quadratic penalties (i.e. applying LQ framework to the states and controls achieved with different objective functions) *LQJ*. This presents an LQ-normalized and thus more comparable basis for the functions, but also—as expected—indicates LQ results to be the best;
- The above-mentioned weighted variance (*WVar*), which makes it possible to compare the objective functions in terms of the volatility in state and control time series.

The first column in Table 2 contains the objective function values for the uncontrolled solution, which uses the initial states of the corresponding systems and is intended only for a comparison with the optimal results. The second column contains optimal objective values as calculated by the OPTCON2 algorithm. The fifth column gives the objective values as calculated by Differential Evolution (DE) using a standard objective function (LQ framework) demonstrating that DE converges to basically the same solution as OPTCON but taking considerably more time (‘cpu’). The third column gives the objective values as calculated by DE using the LMS objective function (LMS), while the fourth column contains the results for the objective function with absolute values (ABS). The last two columns state the results for the cubic (CUB) and quartic (QUART) loss functions.

In terms of the LQ-normalized objective function values (LQJ), all alternatives except ABS show more or less similar results. Certainly, the scenario with LQ function to be minimized demonstrates the best result, but this simply indicates that the DE algorithm does its work well. If one had normalized the results not on the quadratic but, e.g., on the quartic objective function, there is no doubt that the QUART result would have been the lowest. However, taking the volatility (*WVar*) into account, there are significant differences in performance. While a higher power in the objective function leads to a reduction of the volatility, the LMS and the ABS approaches result in much greater instability of the Austrian economy.

Hence, the actual trade-off one is facing is to find an optimum within the shortest computational time (optimize the quadratic function by the OPTCON algorithm as reported by the second column in Table 2) or use a more sophisticated objective function accounting for additional policy objectives. In this particular case, the alternatives applying deviations from the targets a power above two seem most promising. The larger the power, the smaller the variance in states and controls over time. Thus, we find that using larger exponents on deviations in states and controls one can better restrict volatility in macroeconomic variables while ensuring that the system reacts to the external shocks and minimizes its deviations from the targets given.

Note at this point that this study does not argue that, for example, the cubic loss function is always better than the quadratic one. Instead, we illustrate that the loss functions with higher exponents better enable to account for additional constraints such as volatility in the states and control variables over time. Imagine that the volatility (measured by the weighted volatility *WVar*) would be added as a penalty to the objective (loss) function. Then this penalty could result in the quartic or cubic functions outperforming the standard quadratic one. Elsewise, one could introduce some upper threshold on the volatility, which could again indicate in favor of the alternative loss function specifications. Clearly, more research on this matter first has to be done to make any concrete numerical suggestions. All we want to say is that optimal control problems may be far more complex (i.e. requiring broader set of loss functions to be considered and/or additional constraints to be included) than to be solved by the ‘one size fits all’ LQ approach.

## Discussion and conclusion

In this paper we compare alternative forms of objective function specifications in the context of nonlinear dynamic optimal control problems experiencing a one-time exogenous shock. Given that the alternative specifications are not necessarily well behaved, we optimize them not by means of a linear-quadratic optimization technique but using an evolutionary algorithm, namely Differential Evolution.

Applying the alternative functional forms on a new small-scale model of the Austrian economy (ATOPT), we find that the traditional (quadratic) objective function does not perform as well as its alternatives (using a power greater than two) in restricting the volatility of the resulting optimal paths of the corresponding states and controls. The latter, in fact, has an important side effect on macroeconomic instability, which a policy maker tends to avoid. This serves as further evidence that the (historical) dominance of the quadratic tracking form objective function specification present in the literature, due to its convenience in applying LQ framework^11^ in finding its optimum, does not automatically imply that this quadratic form is an optimal choice for various problems and model scenarios to be considered. Hence, a more thorough selection of a suitable objective function accounting for multiple objectives pursued by policy makers is necessary.

The latter argument, in fact, leads us to a more general problem. For many decades various optimization problems have been simplified (from any additional restrictions) to fit some existing optimization frameworks (and their assumptions), so as to allow fast and guaranteed convergence. Nowadays, we observe the opposite trend: thanks to the recent advances in computing technology, one could develop more time consuming methods by either modifying existing optimization algorithms or by offering novel and much more flexible (derivative free) nature-inspired optimization methods, like the heuristics, which can allow for as many additional restrictions as one needs. The example of the volatility in the state and control variables of the ATOPT model is just one example. For more examples one can read Blueschke et al. (2013) (asymmetry of the objective function) or Blueschke et al. (2015) (non-negative values of control variables). This study demonstrates that alternative functional forms of an objective (loss) function can easily be applied when using the evolutionary heuristic approach for solving optimal control problems. Moreover, it is illustrated that the quadratic functional form is not the ‘perfect hammer’ accounting for different objectives one has. Effectively, this study calls for some sort of meta-theory, which helps to choose among different policy objectives. Including an additional penalty for volatility, or some kind of risk aversion, could be an example of such a more sophisticated way of choosing an optimal policy. The formulation of more exact functional forms, which better address specific objectives one is willing to balance remains, from our point of view, problem-specific so far and is left for further research.

## Footnotes

1 The trade-off between flexibility and higher computational demand is well known for heuristic optimization. For a concise discussion of the matter see Gilli and Schumann (2014).

2 To the best of our knowledge, the only exception is c (Bass and Webber 1966).

3 See, for example, Luus et al. (2001) for the application of an absolute values based objective function to optimal control problems.

4 The first three equations are estimated by OLS. The last equation is an identity.

5 The unemployment rate can also be considered as a proxy for the economic situation and the output.

6 Which is a reasonable assumption for the small, trade-dependent Austrian economy.

7 However, the exercise can be easily extended to model an unexpected shock. For this reason, one would have to apply the DE algorithm twice: first, before the shock is known on the entire period of interest; and second, once the shock takes place—at the period between the shock and the remaining part of the calculation period. Again, given that the focus of the present paper is the specification of the objective function to be applied and not the economic conclusion the ATOPT model provides, we choose a simpler ex-ante option.

8 An alternative would have been to measure the coefficient of variation (CV), which standardizes the variances by the corresponding averages making it easy to compare between different variables and aggregate them into one single indicator. However, given that some of our variables have averages close to zero, this biases the result making CV not applicable.

9 We have also tested the possibility of applying the median of squares to both the states and the controls, but the resulting dynamics of the variables under consideration become even more volatile and intractable in such a case and we did not proceed in considering it further. Results are available on request.

10 Optimization of linear systems with Gaussian noise under a quadratic objective function.

11 Indeed, Cukierman (2002), p. 23 describes the quadratic (penalization) function as the one being ‘chosen mainly for analytical convenience rather than for descriptive realism’.
